# Simulating workload reduction with an AI-based prostate cancer detection pathway using a prediction uncertainty metric

**DOI:** 10.1007/s00330-025-11727-6

**Published:** 2025-06-07

**Authors:** Stefan J. Fransen, Joeran S. Bosma, Quintin van Lohuizen, Christian Roest, Frank F. J. Simonis, Thomas C. Kwee, Derya Yakar, Henkjan Huisman

**Affiliations:** 1 Department of Radiology, University Medical Center Groningen, Groningen, The Netherlands; 2 Radboud University Medical Center, Diagnostic Image Analysis Group, Nijmegen, The Netherlands; 3 TechMed Centre, Technical University Twente, Enschede, The Netherlands

**Keywords:** Artificial intelligence, Computer-assisted diagnoses, Prostatic neoplasms, Workload, Magnetic resonance imaging

## Abstract

**Objectives:**

This study compared two uncertainty quantification (UQ) metrics to rule out prostate MRI scans with a high-confidence artificial intelligence (AI) prediction and investigated the resulting potential radiologist’s workload reduction in a clinically significant prostate cancer (csPCa) detection pathway.

**Materials and methods:**

This retrospective study utilized 1612 MRI scans from three institutes for csPCa (Gleason Grade Group ≥ 2) assessment. We compared the standard diagnostic pathway (radiologist reading) to an AI-based rule-out pathway in terms of efficacy and accuracy in diagnosing csPCa. In the rule-out pathway, 15 AI submodels (trained on 7756 cases) diagnosed each MRI scan, and any prediction deemed uncertain was referred to a radiologist for reading. We compared the mean (meanUQ) and variability (varUQ) of predictions using the DeLong test on the area under the receiver operating characteristic curves (AUROC). The level of workload reduction of the best UQ method was determined based on a maintained sensitivity at non-inferior specificity using the margins 0.05 and 0.10.

**Results:**

The workload reduction of the proposed pathway was institute-specific: up to 20% at a 0.10 non-inferiority margin (*p* < 0.05) and non-significant workload reduction at a 0.05 margin. VarUQ-based rule out gave higher but non-significant AUROC scores than meanUQ in certain selected cases (+0.05 AUROC, *p* > 0.05).

**Conclusion:**

MeanUQ and varUQ showed promise in AI-based rule-out csPCa detection. Using varUQ in an AI-based csPCa detection pathway could reduce the number of scans radiologists need to read. The varying performance of the UQ rule-out indicates the need for institute-specific UQ thresholds.

**Key Points:**

***Question*** *AI can autonomously assess prostate MRI scans with high certainty at a non-inferior performance compared to radiologists, potentially reducing the workload of radiologists*.

***Findings*** *The optimal ratio of AI-model and radiologist readings is institute-dependent and requires calibration*.

***Clinical relevance*** *Semi-autonomous AI-based prostate cancer detection with variational UQ scores shows promise in reducing the number of scans radiologists need to read*.

**Graphical Abstract:**

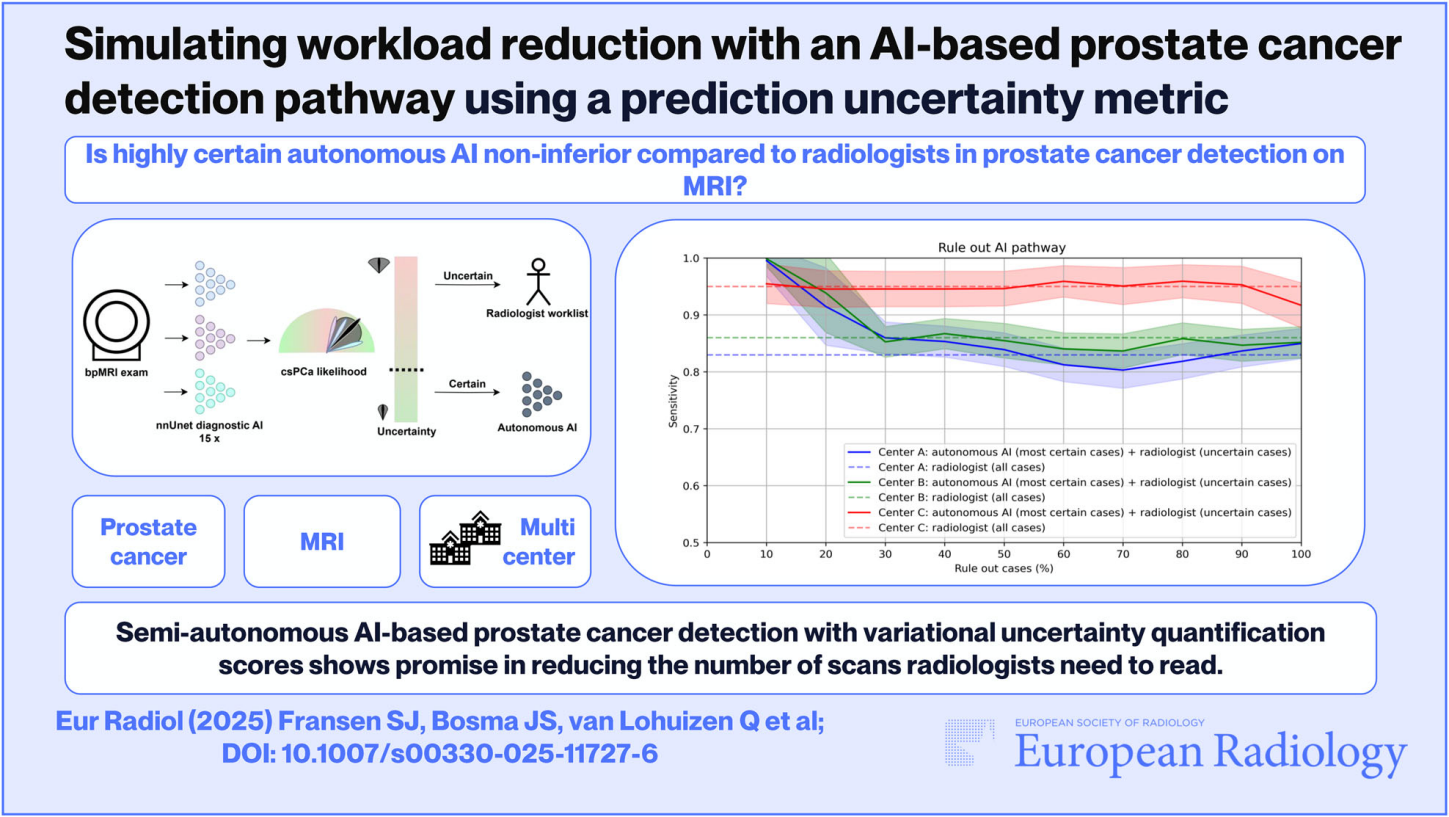

## Introduction

Prostate MRI and MRI-targeted biopsies are acknowledged as precise and cost-effective techniques for detecting clinically significant prostate cancer (csPCa) [1, 2]. However, the increasing frequency of MRI scans in this pathway has led to a higher workload for radiologists, raising sustainability concerns [3].

Artificial Intelligence (AI) models can potentially reduce radiologists’ workloads and enhance detection efficiency, as showcased in, for example, csPCa detection [4], tuberculosis screening [5], and mammography screening [5–7]. Recent advancements in AI models have demonstrated near-expert-level accuracy in diagnosing csPCa [8–10]. Such AI models are primarily studied to provide radiologists with decision support. Although this decision support could provide radiologists with an easy-to-assess second opinion, it does not reduce the number of cases a radiologist has to read. To improve workflow efficiency and meet rising demands, a degree of AI automation is necessary. Emerging semi-autonomous AI models represent a promising solution [6, 7]. For example, a study in mammography screening illustrated the benefits of semi-autonomous AI support in reducing the workload of radiologists [7].

Rule out AI uses a threshold on uncertainty quantification (UQ) to indicate cases that can be automatically reported [6, 7, 11]. AI-based UQ mimics human uncertainty [11]. The performance of AI in cases with a high UQ (i.e., uncertain cases) has been shown to significantly decrease [11]. Thresholding AI UQ allows the automation of the selection of uncertain cases for referral to human experts, while other cases could follow an autonomous workflow. This workflow is referred to as rule out. Only one previous study has described a semi-autonomous AI-based rule-out pathway for csPCa detection [11]. They produced a UQ score by calculating the variance within model ensembles, where the value represents the agreement among models. Another UQ method to consider could be the mean of a model ensemble, where certain cases approach extreme values near zero or one. Another underexplored aspect is the evaluation of the potential reduction in a radiologist’s workload [11].

We hypothesize that ruling out AI can reduce the radiologists’ workload, defined as the total number of prostate MRIs they need to assess. We set up a simulation study to investigate two aspects: the first was to compare the diagnostic performance of different UQ metrics to rule out prostate MRI scans, and the second was to investigate the potential radiologist’s workload reduction in a semi-autonomous AI-based csPCa detection pathway with prediction uncertainty.

## Materials and methods

### Data

Retrospective, consecutive, multicenter data were used with approval from local institutional ethics committees, who waived the need for informed consent (University Medical Center Groningen (center A): METc-2018/597, Martini Ziekenhuis (center B): MEC-2019-056, Trondheim University Hospital (center C): 2017/576). Patients who underwent MRI assessment for csPCa detection were included between November 2014 and November 2021 at center A, June 2014 and February 2020 at center B, and March 2015 and December 2017 at center C. Included patients had a clinical suspicion of csPCa, no prior csPCa treatment, and an MRI examination containing at least an axial T2 weighted scan, a high *b*-value (≥ b1400) diffusion-weighted image (DWI), and an apparent diffusion coefficient map (ADC). Patients with PI-RADS ≥ 3 without follow-up biopsy or with preprocessing errors in the algorithm were excluded. Expert radiologists (center A: 8–11 years of experience, center B: 11 years of experience, and center C: 2–7 years of experience) scored MRI scans on prostate imaging reporting and data system (PI-RADS) findings. MRI findings of PI-RADS ≥ 3 were followed by targeted biopsies in combination with systematic biopsies, in which a Gleason Grade Group (GGG) of ≥ 2 was considered as csPCa ground truth [12]. Following the PI-RADS guidelines, patients rated as PI-RADS 1 or 2 did not undergo a follow-up biopsy, being considered negative due to the high negative predictive value of PI-RADS [2, 12, 13]. Parts of the dataset have been previously used in prior articles with different purposes: deep learning csPCa detection [8, 9, 14], radiomics csPCa detection [15], csPCa biopsy detection efficacy [16], and csPCa follow-up detection [17].

### AI model

A previously developed state-of-the-art AI model for csPCa detection was used in this study [8]. An extensive description of the algorithm (e.g., model generalization and training processes) is provided in the article by Bosma et al [8]. The algorithm is publicly available (https://grand-challenge.org/algorithms/bpmri-cspca-detection-report-guided-annotations/). The algorithm leverages the nnU-Net framework, which dynamically adjusts its configuration to determine the architecture for a given input dataset [18]. The model is trained to predict csPCa lesions using an axial T2-weighted scan, high *b*-value (> b1400) DWI, and an ADC as input. As preprocessing, the model takes a center crop of 80.0 mm × 80.0 mm × 72.0 mm and does resampling to a resolution of 0.5 mm × 0.5 mm ×3.6 mm/voxels. The model was trained on 7756 prostate MRI examinations (Siemens MRI scanner) with segmentations for csPCa lesions from Radboud University Medical Center. The training was performed with five-fold cross-validation (i.e., 6205 training cases and 1551 validation cases) and three restarts, providing 15 independent submodels yielding heatmaps with voxel-level likelihoods with values of 0 for healthy tissues and 1 for malignant tissues. The combined predictions of the submodels are referred to as an ensemble, and the average of the 15 submodels was used as the final prediction.

### Rule out pathway

**Fig. 1:**
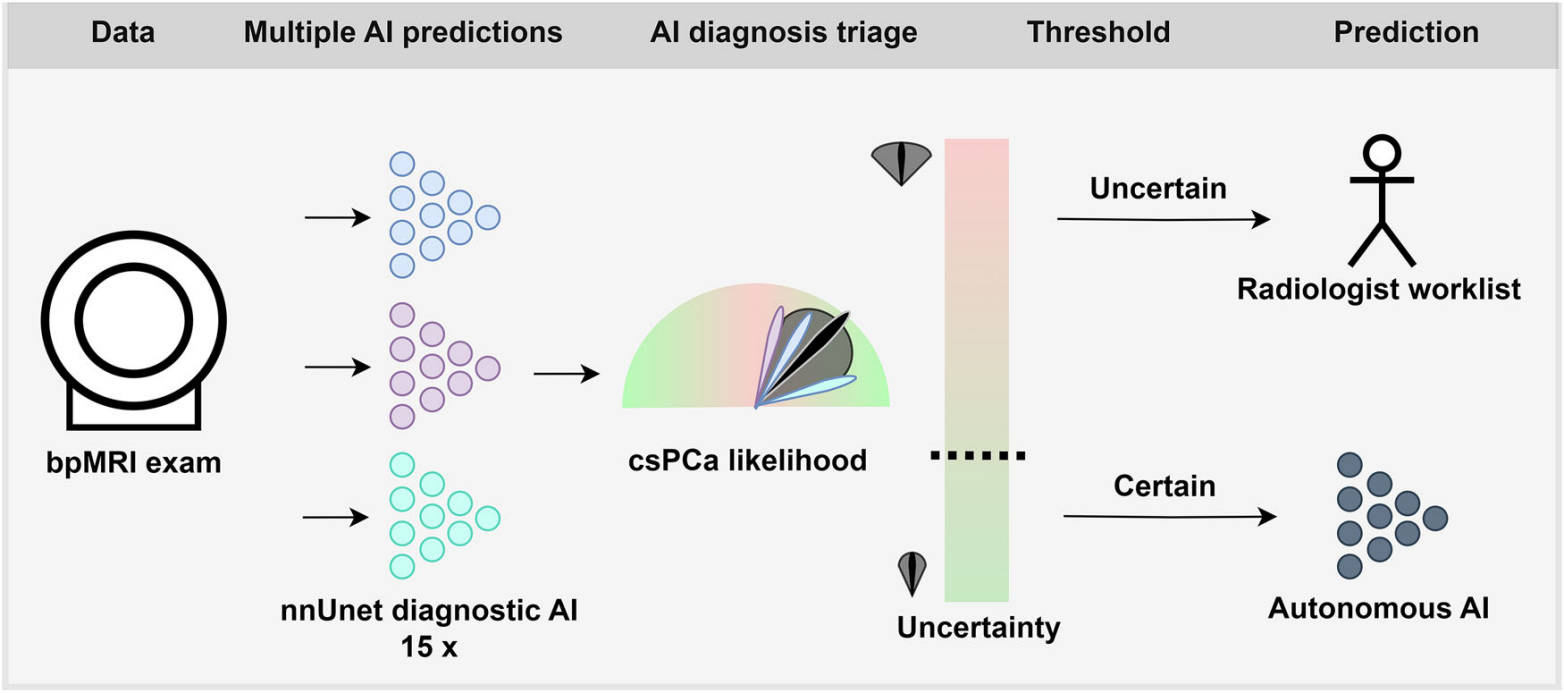
Schematic representation of the proposed AI-based rule-out csPCa detection pathway. In the rule-out pathway, multiple diagnostic AI submodels assessed a prostate MRI scan. Each of these submodels was trained separately and provided a different prediction score. Based on the difference in prediction scores, a UQ score was calculated. These UQ scores allowed for (1) ranking cases based on uncertainty and (2) ruling out certain cases with high diagnostic performance, allowing for automated AI model reading. The average of the submodel predictions was used as the combined prediction from the AI model

Rule out is implemented by thresholding the AI UQ score and referring a case to either an autonomous AI-based or a radiologist interpretation. Figure 1 shows a schematic representation of the workflow of the proposed semi-autonomous AI-based rule-out csPCa detection pathway. Both cases with and without csPCa were considered to reduce the radiologist’s workload.

### UQ

**Fig. 2:**
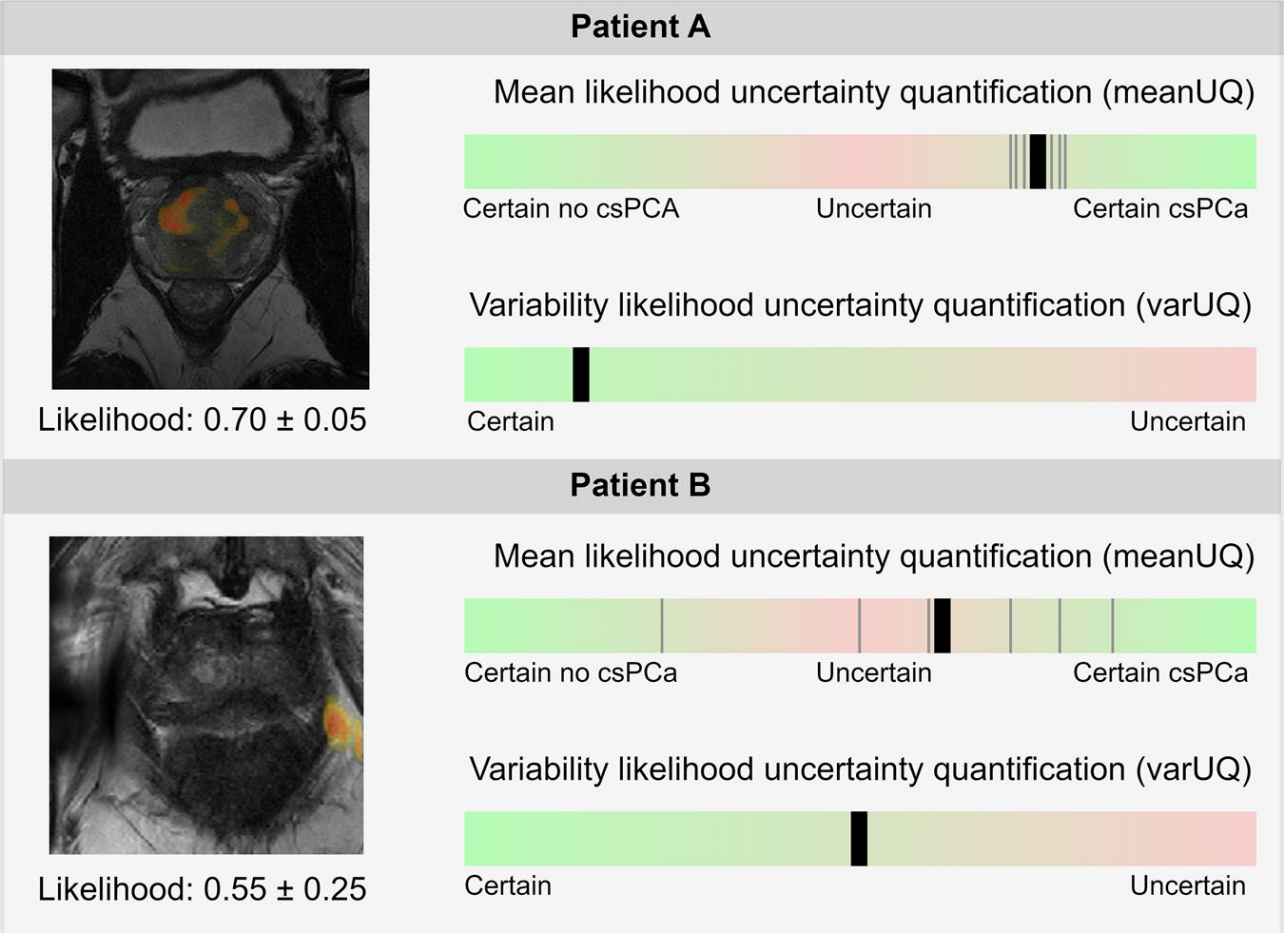
Illustrations of meanUQ and varUQ metrics to assess the certainty of AI diagnosis. In both illustrations, the metrics offer slightly different indications regarding the level of certainty in the AI diagnosis, indicated by the bold line on the certainty bar. In patient A (57-years-old, PSA of 9.6 µg/L, PI-RADS 5, and GGG 2), the AI predicts a mean likelihood of prediction of 0.70 for csPCa presence with a variability of 0.05. In patient B (51-years-old, PSA of 2.5 µg/L, PI-RADS 4, and GGG 1), the AI predicts a mean likelihood of 0.57 for csPCa presence with a variability of 0.25

Two metrics for UQ-based rule-out were studied: mean uncertainty quantification (meanUQ) and variational uncertainty quantification (varUQ). Recently, varUQ was proposed as a UQ metric, but a comparison with the common mean of prediction ensembles (meanUQ) is lacking. The calculation of both metrics used the output of the AI model, yielding heatmaps with voxel-level likelihoods of malignant tissues. The meanUQ metric uses the mean of the ensemble of patient-level predictions. Mean prediction scores close to 0 are certain benign, close to 0.5 are considered uncertain, and close to 1 are considered certain csPCa. To reflect this, meanUQ was defined as the absolute value of the mean patient-level prediction subtracted by 0.5. (e.g., low meanUQ scores are uncertain and high meanUQ scores are certain). The varUQ metric was defined as the standard deviation over the patient-level predictions [11]. Cases with a low varUQ score are certain, whereas cases with a high varUQ score indicate the AI was uncertain, and cases were susceptible to low AI performance. Figure 2 shows an example of an uncertain and certain prediction using meanUQ and varUQ.

### Experiments and statistical analysis

In two experiments, we investigated the diagnostic performance of an AI-based rule-out csPCa detection pathway and the potential radiologists’ workload reduction. The first experiment compared the efficacy of meanUQ and varUQ to rule out patients with a certain AI diagnosis. The second experiment used the best UQ from experiment 1 to determine the reduction in the radiologists’ workload while maintaining the diagnostic performance of the current radiologist pathway. In both experiments, the statistical significance threshold was defined as 0.05.

#### Experiment 1: rule out with meanUQ or varUQ

The first experiment compared the efficacy of meanUQ and varUQ to rule out cases. The rule-out threshold for both meanUQ and varUQ was determined using Youden’s Index by selecting the UQ threshold that maximizes the detection of csPCa at the minimal cost of false positives [19]. The diagnostic performance of both UQs was compared across each institute separately to account for institutional variations. Diagnostic performance was measured as the area under the receiver operating characteristic curve (AUROC). The DeLong test was used to statistically compare both AUROCs in RStudio version 4.2.0 using the pROC package [20, 21]. The DeLong test compares two ROC curves by considering the implicit correlation that both curves are calculated from the same test cases [21]. In addition, the number of certain cases found with varUQ and meanUQ was compared, and the percentage of certain varUQ cases that were also identified as certain cases using meanUQ was calculated.

#### Experiment 2: AI-based rule out the csPCa detection pathway

The second experiment compared the simulated AI-based rule-out pathway with the radiologist pathway to determine the reduction in the radiologists’ workload while maintaining the diagnostic performance of the current radiologist pathway. The AI-based rule-out pathway embedded the best-performing UQ metric (based on AUROC) from experiment 1. The diagnostic performances were compared at multiple levels of workload reduction by increasing the UQ threshold as a percentage of cases ruled out by 10% increments (10%, 20%, and up to 100% autonomous AI model reading). The sensitivity of the AI-based rule-out pathway was compared with the respective radiologists’ sensitivity during clinical practice. In line with Dvijotham et al, non-inferiority was established at superior sensitivity at a non-inferior specificity operating point [18]. The sensitivity of the AI was calculated at two non-inferior specificity operating points that were calculated by subtracting the margins of 0.05 and 0.10 from the corresponding radiologists’ specificity; the margins represent the trade-off between more biopsies and reduced radiologists’ workload (i.e., more biopsies are accepted if the radiologists’ workload can be reduced to create a sustainable csPCa detection pathway). We did not perform a non-inferiority analysis on radiologists’ sensitivity because this would increase false-negative patients by an accepted margin, leading to long-term patient harm because a proportion of csPCa patients would not receive treatment. Nonetheless, we did include an overview of the sensitivities at fixed specificity operation points in the Supplementary Materials. The radiologists’ sensitivities were calculated based on the readings during clinical practice (PI-RADS ≥ 4) compared with the biopsy findings [8]. Statistical analysis was performed using a permutation test with 10.000 permutations, in which the patient was the resampling unit.

## Results

### Data

**Fig. 3:**
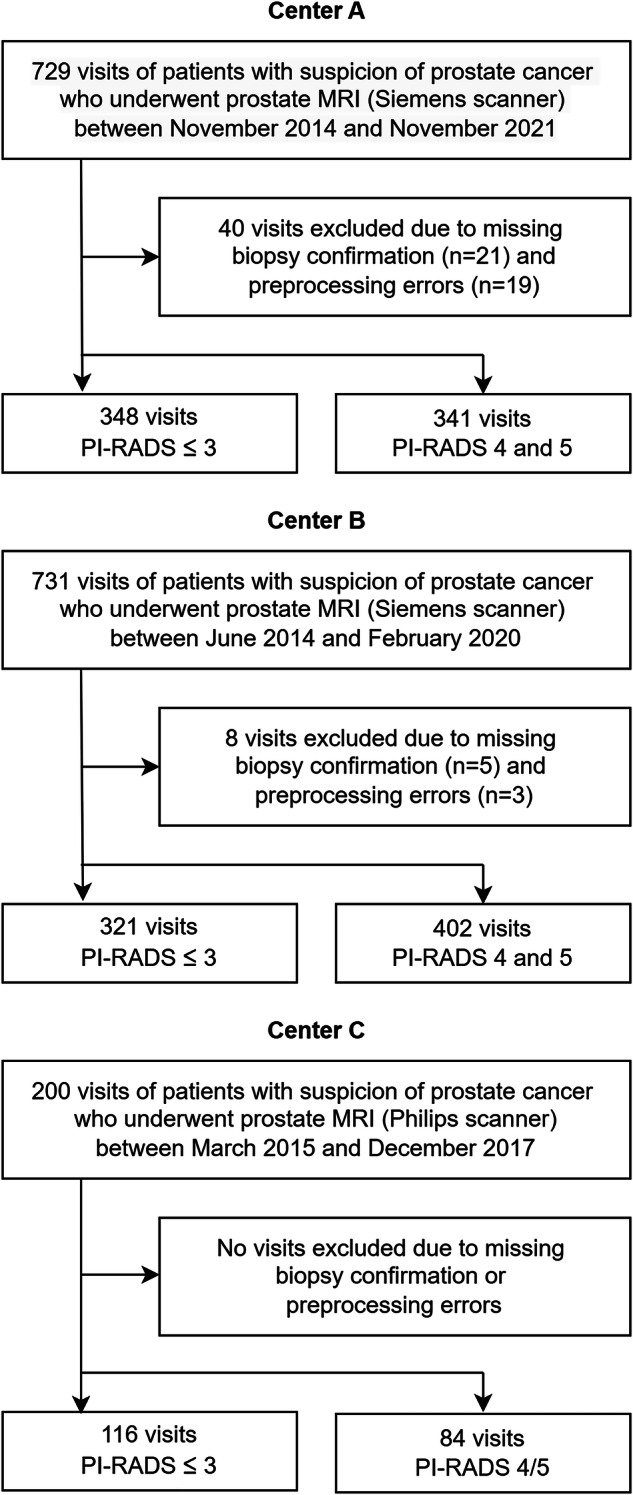
This figure shows the patient selection. This study included multicenter data from centers A, B, and C to test the proposed AI-based rule-out csPCa detection. A separate dataset was used to train a state-of-the-art csPCa detection AI model. This detection AI model was used to externally validate the AI-based rule-out csPCa detection pathway on data from centers A, B, and C

A total of 1612 patients’ visits were included with an MRI scan prostate cancer diagnosis as first visit and active surveillance: 689 visits of 625 patients from center A, 723 visits of 571 patients from center B, and 200 visits of 200 patients from center C (Fig. 3). Patient characteristics and radiologists’ performances per institute are shown in Table 1. The median patient age was 68 years, and the median PSA level was 8 µg/L. The median age and PSA in center A were 69 years and 7.9 µg/L, in center B 68 years and 8.5 µg/L, and in center C 66 years and 7.3 µg/L. The radiologists’ sensitivity at each institute was above 80%, and their specificity was above 50%. The scan parameters are shown in Table 2. All scans were acquired during a standard clinical routine, in center A on 1.5-T (21%, *n* = 145) and 3-T (79%, *n* = 544) MRI scanners from Siemens (84%, *n* = 578) (Siemens Medical Solutions) and Philips (16%, *n* = 111) (Philips Healthcare), in center B on 1.5-T (7%, *n* = 52) and 3-T (93%, *n* = 671) MRI scanners from Philips (100%, *n* = 723), and in center C all on 3-T MRI scanners from Siemens (100%, *n* = 200). The imaging protocol included T2-weighted axial imaging, high *b*-value (> b1400) DWI, and an ADC, following the recommendations from PI-RADS [22].

**Table 1:** Patient characteristics and institute performances of data from centers A, B, and C

Institute	Inclusion (patients’ visits)	Clinical variables (median and IQR)		GGG		Radiologists performance	
		Age (year)	PSA (µg/L)	1	*≥* *2*	Sensitivity	Specificity
Center A	689	69 (10)	7.9 (5.5)	75% (*n* = 514)	25% (*n* = 175)	83%	58%
Center B	723	68 (9)	8.5 (6.2)	75% (*n* = 542)	25% (*n* = 181)	86%	54%
Center C	200	66 (9)	7.3 (7.3)	72.5% (*n* = 145)	27.5% (*n* = 55)	95%	78%

The radiologists’ performance was calculated by comparing PI-RADS 4 and 5 and GGG ≥ 2 as csPCa

*IQR* interquartile range

**Table 2:** MRI scan parameters of the three institutes

Characteristic	Center A *n* = 689	Center B *n* = 723	Center C *n* = 200
T2-weighted axial acquisition			
In-plane resolution (mm/voxel)	0.37 ± 0.15	0.35 ± 0.02	0.51 ± 0.02
Slice thickness (mm/voxel)	3.08 ± 0.25	3.07 ± 0.26	3.00 ± 0.04
Averages	3.06 ± 0.65	1.06 ± 0.23	2.94 ± 0.25
DWI			
In-plane (mm/voxel)	1.42 ± 0.40	1.38 ± 0.06	1.99 ± 0.10
Slice thickness (mm/voxel)	3.34 ± 0.53	3.14 ± 0.52	3.01 ± 0.11
Averages	b0: 6.07 ± 2.39 b50: 1.84 ± 1.02 b400: 5.17 ± 1.21 b500: 4.10 ± 1.29 b800: 6.39 ± 2.52 b1000: 6.79 ± 1.88	b0: 3.04 ± 0.35 b50: 2.98 ± 0.26 b400 2.97 ± 0.24 b500: 3.98 ± 0.14 b800: 3.04 ± 0.34 b1000: 3.98 ± 0.14	b0: 8 b50: 8 b400: 8 b800: 8 b1000: 8
Computed *b*-value (s/mm^2^)	b1400 (*n* = 643) b1600 (*n* = 52) b2000 (*n* = 3)	b1400 (*n* = 723)	b1400 (*n* = 200)
Acquired *b*-values (s/mm^2^)	(50, 500, 1000) ×416 (0, 100, 400, 800) ×84 (50, 400, 800) ×50 Other ×139	(0, 50, 400, 800) ×643 (0, 500, 800, 1000) ×47 (0, 50, 500, 800) ×28 Other ×5	(0, 50, 400, 800) ×198 (0, 1000) ×2

The most common acquired *b*-values and *b*-value averages are reported

### Experiment 1: comparing meanUQ and varUQ

Although non-significant, certain cases selected with varUQ obtained a higher performance than those selected with meanUQ (*p* = 0.9 in center A; *p* = 0.08 in center B; *p* = 0.4 in center C). For the meanUQ, the Youden index resulted in 459 certain cases (66.61%) at a threshold of 0.32 for center A, 475 certain cases (65.70%) at a threshold of 0.29 for center B, and 125 certain cases (62.50%) at a threshold of 0.31 for center C. The meanUQ-pathway performances were 0.72 AUROC at center A, 0.68 AUROC at center B, and 0.91 AUROC at center C. For the varUQ, the Youden index resulted in 224 certain cases (32.51%) at a threshold of 0.094 for center A, 247 certain cases (34.16%) at a threshold of 0.087 for center B, and 62 certain cases (31.00%) at a threshold of 0.037 for center C. The varUQ-pathway performances were 0.72 AUROC at center A, 0.78 AUROC at center B, and 0.95 AUROC at center C. A high number of certain cases with varUQ were also identified as certain cases using meanUQ: *n* = 207 (92.41%) in center A, *n* = 215 (87.04%) in center B, and *n* = 50 (80.64%) in center C. An overview of different performance metrics is provided in the Supplementary Materials.

### Experiment 2: workload reduction

**Fig. 4:**
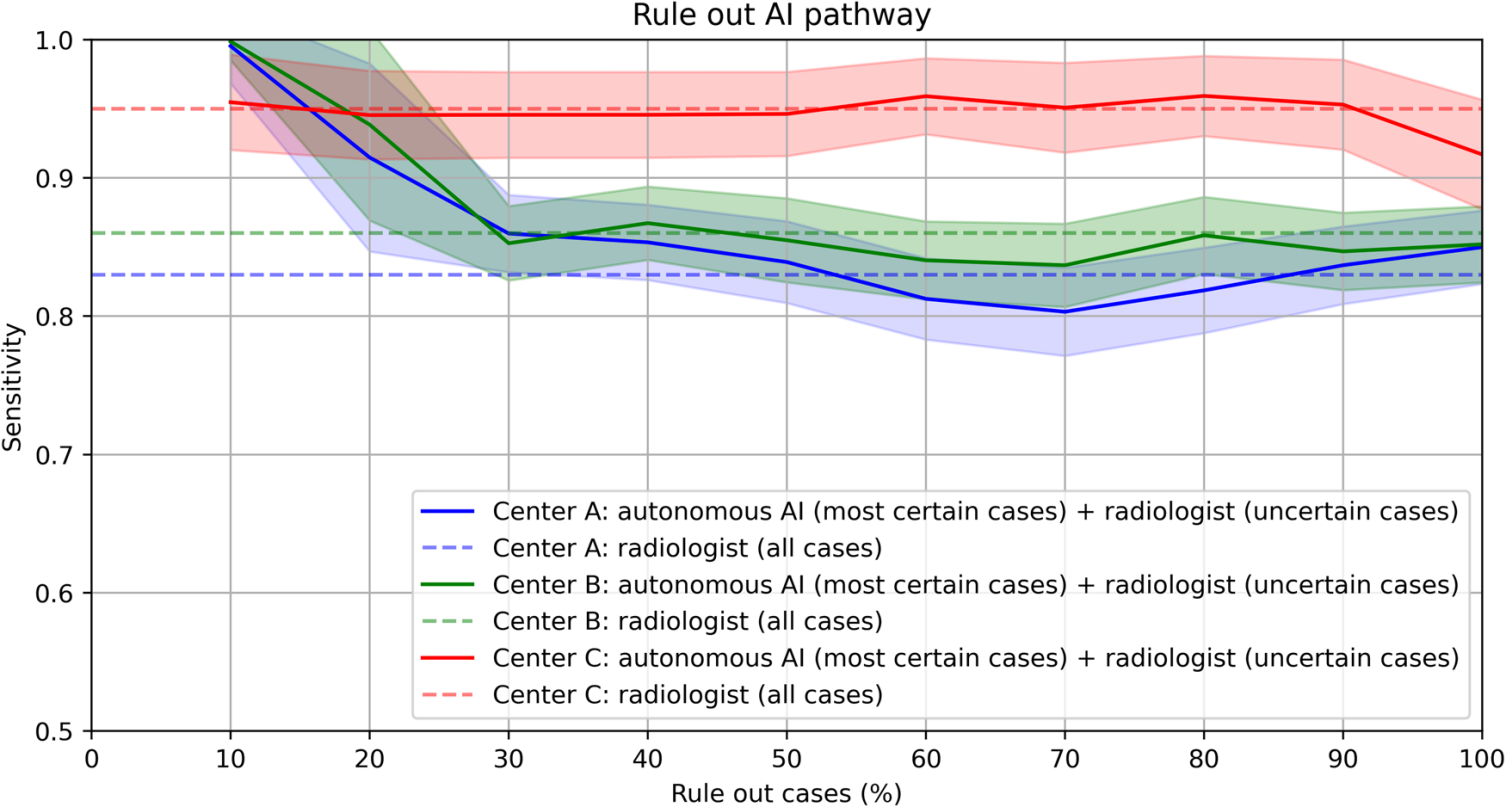
This figure shows the rule-out csPCa detection performances vs the current radiologists’ performances in each institute. The full lines show the combined sensitivity of certain cases assessed by AI plus uncertain cases assessed by the radiologist. Here, the percentage of cases that are completely autonomously read indicates the percentage of autonomous AI model readings without the involvement of a radiologist. The dotted lines show the sensitivity of the radiologists during clinical practice, where all cases are read by radiologists (center A: 0.83 sensitivity, 0.58 specificity; center B: 0.86 sensitivity, 0.54 specificity; center C: 0.95 sensitivity, 0.78 specificity). The sensitivities of the full lines are calculated at the specificity operating point of the corresponding radiologists at each center, reading the same subset of cases minus the non-inferiority margin of 0.10. The shaded area represents the standard deviation from the mean performance

**Fig. 5:**
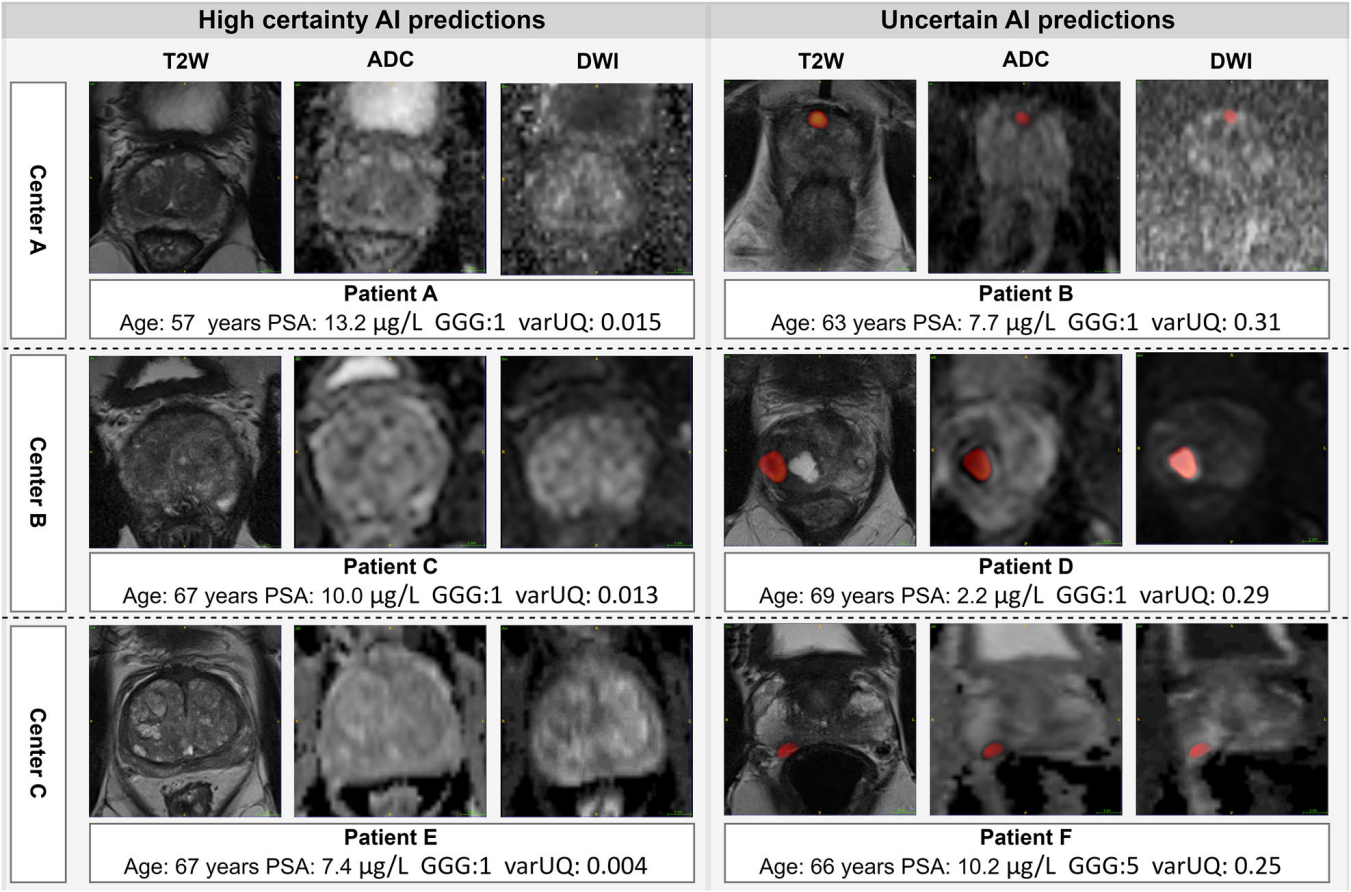
This figure shows the most certain and uncertain AI predictions based on varUQ of each center in the test set. In the most certain AI predictions, the AI was certain no lesions were present. In the uncertain cases, the AI was uncertain about the presence of a lesion

VarUQ-based rule out resulted in a statistically significant workload reduction of 20% in center A and 10% in center B (*p* < 0.05) at a reduced specificity margin of 0.10, see Fig. 4 and Table 3. At a 10% workload reduction in center C, a non-significant increase in sensitivity was observed at a specificity margin of 0.10 (*p* = 0.376). At a reduced specificity margin of 0.10, the sensitivities of the radiologists (center A: 83%, center B: 86%, and center C: 95%) were maintained at an accepted specificity of 48% in center A, 44% in center B, and 68% in center C. The percentage of PI-RADS ≥ 3 lesions was 77% (*n* = 103) in center A at 20% workload reduction, 67% (*n* = 49) in center B at 10% workload reduction, and 15% (*n* = 3) in center C at 10% workload reduction. At a stricter non-inferiority specificity margin of 0.05, the added value of semi-autonomous AI-based csPCa diagnosis was not significant (*p* = 0.153 for 20% workload reduction in center A, *p* = 0.243 for 10% workload reduction in center B, and *p* = 0.463 for 10% workload reduction in center C). Figure 5 shows the most certain and uncertain AI predictions of each center. An overview of the sensitivities at fixed specificity operation points is provided in the Supplementary Materials.

**Table 3:**
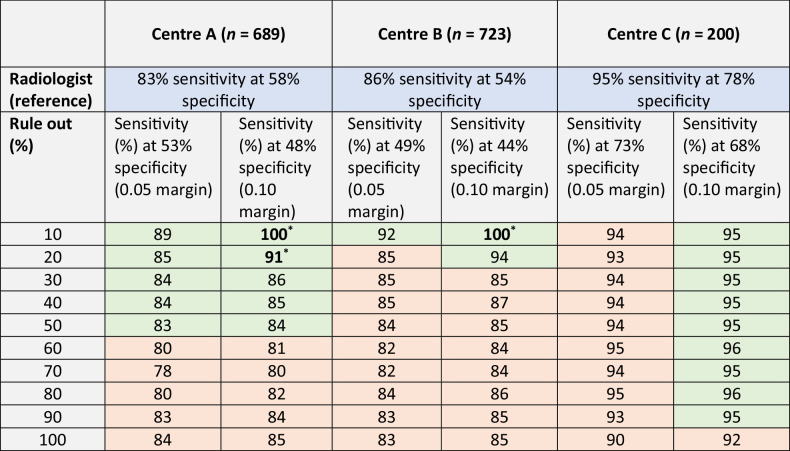
This table shows the performance of the semi-autonomous AI-based rule-out pathway vs the radiologists’ pathway performance at the non-inferior specificity margins of 0.05 and 0.10

A statistically significant sensitivity improvement is represented in bold with *p < 0.05

In blue, the radiologists’ sensitivity performances are shown (center A: 83% sensitivity and 58% specificity, center B: 86% sensitivity and 54% specificity, and center C: 95% sensitivity and 78% specificity). In green, the proposed AI-based rule-out pathway sensitivity performances are shown that maintain or improve the sensitivities of the radiologists up to the first reduced sensitivity. In red, the proposed AI-based rule out pathway sensitivity performances are shownwith reduced sensitivities compared to the radiologists. For example, at a 0.05 non-inferiority margin, the performance of 10% AI model readings in center A is 89% sensitivity at 53% specificity vs the radiologists' performance of 83% sensitivity and 58% specificity. A statistical significant sensitivity improvement is represented in bold with *p < 0.05

## Discussion

This simulation study on external data investigated the feasibility of AI-based csPCa detection to potentially reduce the radiologist’s workload. A radiologist’s workload reduction might be realized by enabling AI models to independently manage high certainty and straightforward prostate MRI scans and reserving ambiguous ones for human experts. The workload reduction of the proposed pathway was institute-specific, varying between 20% at center A, 10% at center B, and no reduction at center C at a specificity margin of 0.10. Semi-autonomous AI model readings bring the risk of more biopsies, but a threshold customizable by the user could enable centers to set the AI model’s threshold at their preferred level of workload reduction and diagnostic performance (e.g., centers with a shortage of radiologists might want more autonomous AI readings and allow more biopsies compared to centers without sustainability issues in the current radiologists’ csPCa detection pathway). Our results indicate that the current AI quality of csPCa detection shows the potential to help reduce the radiologist’s workload and offer a more sustainable csPCa detection pathway solution. With the continuing increase in AI quality, the effect of AI-based rule-out csPCa detection on radiologist workload reduction might increase over time.

To the best of our knowledge, this is the first study that investigated the potential radiologist’s workload reduction using rule out in the csPCa detection pathway. Therefore, it is challenging to compare our findings with existing literature. When comparing our findings with existing literature, a study by Alves et al emerged that used the same state-of-the-art csPCa detection algorithm for AI-based csPCa rule out with varUQ [11]. While our study used varUQ to rule out certain cases, it showed that varUQ could identify cases susceptible to AI failure and concluded that future research was needed on multicenter data to assess UQ in optimizing clinical workflows. Our study performed a missing evaluation of the potential radiologist’s workload reduction. Moreover, our study also compared the efficacy of varUQ and meanUQ to rule out patients with a certain AI diagnosis and showed both metrics have a similar performance: a high certainty in meanUQ suggests uniformity in individual predictions, leading to a similarly high certainty in varUQ. When comparing our findings with semi-autonomous AI-based diagnosis in other pathologies with a screening setting, a comparison can be made with a study by Lauritzen et al and a study by Dvijotham et al [5, 7]. Lauritzen et al studied semi-autonomous AI-based mammography assessment in a screening setting [7]. In their study, mammography scans were assessed by an AI tool, and based on a threshold in the AI’s risk score, scans were ruled out to autonomous AI model reading or radiologists’ reading. This semi-autonomous AI-based pathway in mammography screening resulted in a similar diagnostic performance compared with standard screening while reducing the radiologist workload by 63% with a non-inferior sensitivity margin of 0.05 (i.e., in a screening setting, a non-inferior sensitivity margin was used instead of our non-inferior specificity margin). Our study found a more preserved workload reduction for csPCa detection using our proposed semi-autonomous AI-based rule-out diagnosis pathway; the difference might stem from the difference between a screening and diagnosis setting. Our results emphasize the potential of UQ-based rule-out to also optimize clinical detection workflows. A comparison could also be made with a study by Dvijotham et al, investigating AI-based diagnosis with UQ-based rule out to decide between an AI diagnosis or standard clinical workflow for breast cancer and tuberculosis detection. Their study showed that semi-autonomous AI-based diagnosis could reduce the radiologists’ workload for breast cancer and tuberculosis detection [5]. Similar to our study, they found that semi-autonomous AI-based detection performances were institute and manufacturer-specific, emphasizing the need for institute-specific UQ thresholds.

A difference in workload reduction and model performance was observed between different centers. These differences between centers might stem from differences in image quality. Factors affecting image quality are, for example, more DWI averages increasing the signal-to-noise ratio, T2 image slice thickness (e.g., some images exceeded 3 mm slice thickness, exceeding PI-RADS technical specifications [22]), or registration errors between the input images [23]. Differences in model performance could also be attributed to differences between test data and training data, as comparable data would obtain a higher performance. The performances of center C were higher for both the radiologists and AI; this might indicate that these cases had a higher image quality (i.e., a higher number of DWI averages was found), were easier to interpret (i.e., a higher percentage of GGG ≥ 2 was found), or were more comparable to the training data [8].

This study has limitations. The AI model was trained on a single-center training set, which did not include scans from the multicenter external test sets. An overall increase in AI performance may have been achieved by training the AI model on diverse data, making it more robust to different test datasets. Such an increase in AI performance could lead to an even further increased workload reduction using AI with UQ-based rule-out for diagnosing csPCa on MRI scans. However, this would have limited the external validation of our study. Moreover, future studies might also consider adding additional metrics to report AI performance. In addition, the current pathway may have introduced a bias that gave an unfair advantage to the radiologists since their interpretations were indirectly and directly used as ground truth. Another bias in favor of the radiologists is the use of PI-RADS ≥ 4 as the operating point to calculate the radiologists’ sensitivities. These potential biases may have overestimated radiologists’ performance, and at a lower radiologists’ performance, the detection AI may provide better performance than the radiologists, realizing a higher workload reduction. Generalizing the results of this study to different centers and AI algorithms should be done with caution. As performance and workload might change with semi-autonomous AI-based diagnosis, prospective studies are needed to assess the actual radiologists’ workload reduction and performance. Moreover, excluding AI failure cases may have introduced a bias for overestimating the workload reduction if radiologists require more time to assess these cases. Prospective follow-up studies could include a unified tuning strategy of the UQ metric and recalibration with any change in the scanner or drift in the patient population. Furthermore, with continuing efforts to improve AI performance and generalizability, future multicenter studies are needed to reassess the effect of AI-based rule-out csPCa detection on radiologist workload reduction. In addition, the liability and current regulatory framework have to be considered when implementing autonomous AI [24]. Moreover, the latest regulations in the European Union (i.e., AI Act article 14) do not allow autonomous AI in a high-risk (e.g., medical) setting, with only a few exemptions on specific use cases [24]. The value of autonomous AI is not yet proven in the context of prostate MRI, and future investigations are needed as evidence to allow regulations to evolve and match what could be possible.

In conclusion, MeanUQ and varUQ both show promise in identifying cases with a certain AI diagnosis. Using UQ in a semi-autonomous AI-based csPCa detection pathway could reduce the radiologists’ detection workload. The number of certain cases for autonomous AI assessment ruled out by UQ was institute-specific, indicating the need for institute-specific UQ thresholds.

## Supplementary information

Supplementary information

## Abbreviations

ADC: Apparent diffusion coefficient map
AI: Artificial intelligence
AUROC: Area under the receiver operating characteristic curves
csPCa: clinically significant prostate cancer
DWI: Diffusion-weighted image
GGG: Gleason grade group
meanUQ: Mean uncertainty quantification
PI-RADS: Prostate imaging reporting and data system
UQ: Uncertainty quantification
varUQ: Variational uncertainty quantification

## References

[1] Ahmed HU, El-Shater Bosaily A, Brown LC et al (2017) Diagnostic accuracy of multi-parametric MRI and TRUS biopsy in prostate cancer (PROMIS): a paired validating confirmatory study. Lancet 389:815–822. 10.1016/S0140-6736(16)32401-1 28110982 10.1016/S0140-6736(16)32401-1

[2] van der Leest M, Cornel E, Israël B et al (2019) Head-to-head comparison of transrectal ultrasound-guided prostate biopsy versus multiparametric prostate resonance imaging with subsequent magnetic resonance-guided biopsy in biopsy-naïve men with elevated prostate-specific antigen: a large prospective multicenter clinical study (figure presented.). Eur Urol 75:570–578. 10.1016/j.eururo.2018.11.023 30477981 10.1016/j.eururo.2018.11.023

[3] James ND, Tannock I, N’Dow J et al (2024) The Lancet Commission on prostate cancer: planning for the surge in cases. Lancet 403:1683–1722. 10.1016/S0140-6736(24)00651-2 38583453 10.1016/S0140-6736(24)00651-2 PMC7617369

[4] Winkel DJ, Tong A, Lou B et al (2021) A novel deep learning based computer-aided diagnosis system improves the accuracy and efficiency of radiologists in reading biparametric magnetic resonance images of the prostate: results of a multireader, multicase study. Invest Radiol 56:605–613. 10.1097/RLI.0000000000000780 33787537 10.1097/RLI.0000000000000780

[5] Dvijotham K, Winkens J, Barsbey M et al (2023) Enhancing the reliability and accuracy of AI-enabled diagnosis via complementarity-driven deferral to clinicians. Nat Med 29:1814–1820. 10.1038/s41591-023-02437-x 37460754 10.1038/s41591-023-02437-x

[6] Lång K, Josefsson V, Larsson A-M et al (2023) Artificial intelligence-supported screen reading versus standard double reading in the mammography screening with artificial intelligence trial (MASAI): a clinical safety analysis of a randomised, controlled, non-inferiority, single-blinded, screening accuracy study. Lancet Oncol 24:936–944. 10.1016/S1470-2045(23)00298-X 37541274 10.1016/S1470-2045(23)00298-X

[7] Lauritzen AD, Rodríguez-Ruiz A, von Euler-Chelpin MC et al (2022) An artificial intelligence–based mammography screening protocol for breast cancer: outcome and radiologist workload. Radiology 304:41–49. 10.1148/radiol.210948 35438561 10.1148/radiol.210948

[8] Bosma JS, Saha A, Hosseinzadeh M et al (2023) Semisupervised learning with report-guided pseudo labels for deep learning–based prostate cancer detection using biparametric MRI. Radiol Artif Intell 5:e230031. 10.1148/ryai.230031 37795142 10.1148/ryai.230031 PMC10546362

[9] Saha A, Hosseinzadeh M, Huisman H (2021) End-to-end prostate cancer detection in bpMRI via 3D CNNs: effects of attention mechanisms, clinical priori and decoupled false positive reduction. Med Image Anal 73:102155. 10.1016/j.media.2021.102155 34245943 10.1016/j.media.2021.102155

[10] Twilt JJ, van Leeuwen KG, Huisman HJ et al (2021) Artificial intelligence based algorithms for prostate cancer classification and detection on magnetic resonance imaging: a narrative review. Diagnostics (Basel) 11:959. 10.3390/diagnostics11060959 34073627 10.3390/diagnostics11060959 PMC8229869

[11] Alves N, Bosma JS, Venkadesh KV et al (2023) Prediction variability to identify reduced AI performance in cancer diagnosis at MRI and CT. Radiology 308:e230275. 10.1148/radiol.230275 37724961 10.1148/radiol.230275

[12] Turkbey B, Purysko AS (2023) PI-RADS: Where next? Radiology 307:e223128. 10.1148/radiol.223128 37097134 10.1148/radiol.223128 PMC10315529

[13] Itatani R, Namimoto T, Atsuji S et al (2014) Negative predictive value of multiparametric MRI for prostate cancer detection: outcome of 5-year follow-up in men with negative findings on initial MRI studies. Eur J Radiol 83:1740–1745. 10.1016/j.ejrad.2014.06.026 25048979 10.1016/j.ejrad.2014.06.026

[14] Hosseinzadeh M, Saha A, Brand P et al (2022) Deep learning-assisted prostate cancer detection on bi-parametric MRI: minimum training data size requirements and effect of prior knowledge. Eur Radiol 32:2224–2234. 10.1007/s00330-021-08320-y 34786615 10.1007/s00330-021-08320-y PMC8921042

[15] Bleker J, Kwee TC, Rouw D et al (2022) A deep learning masked segmentation alternative to manual segmentation in biparametric MRI prostate cancer radiomics. Eur Radiol 32:6526–6535. 10.1007/s00330-022-08712-8 35420303 10.1007/s00330-022-08712-8 PMC9381625

[16] Krüger-Stokke B, Bertilsson H, Langørgen S et al (2021) Multiparametric prostate MRI in biopsy-naïve men: a prospective evaluation of performance and biopsy strategies. Front Oncol 11:745657. 10.3389/fonc.2021.745657 34722302 10.3389/fonc.2021.745657 PMC8552019

[17] Roest C, Kwee TC, Saha A et al (2023) AI-assisted biparametric MRI surveillance of prostate cancer: feasibility study. Eur Radiol 33:89–96. 10.1007/s00330-022-09032-7 35960339 10.1007/s00330-022-09032-7 PMC9755080

[18] Isensee F, Jaeger PF, Kohl SAA et al (2021) nnU-Net: a self-configuring method for deep learning-based biomedical medical image segmentation. Nat Methods 18:203–211. 10.1038/s41592-020-01008-z 10.1038/s41592-020-01008-z 33288961

[19] Youden WJ (1950) Index for rating diagnostic tests. Cancer 3:32–35 15405679 10.1002/1097-0142(1950)3:1<32::aid-cncr2820030106>3.0.co;2-3

[20] Turck N, Vutskits L, Sanchez-Pena P et al (2011) pROC: an open-source package for R and S+ to analyze and compare ROC curves. BMC Bioinformatics 8:12–77. 10.1007/s00134-009-1641-y 10.1186/1471-2105-12-77 PMC3068975 21414208

[21] DeLong ER, DeLong DM, Clarke-Pearson DL (1988) Comparing the areas under two or more correlated receiver operating characteristic curves: a nonparametric approach. Biometrics 44:837. 10.2307/2531595 3203132

[22] Turkbey B, Rosenkrantz AB, Haider MA et al (2019) Prostate imaging reporting and data system version 2.1: 2019 update of prostate imaging reporting and data system version 2. Eur Urol 76:340–351. 10.1016/j.eururo.2019.02.033 30898406 10.1016/j.eururo.2019.02.033

[23] Hering A, de Boer S, Saha A et al (2024) Deformable MRI sequence registration for AI-based prostate cancer diagnosis. International workshop on biomedical image registration. ACM, Morocco, pp 148–162. 10.1007/978-3-031-73480-9_12

[24] Kotter E, D’Antonoli TA, Cuocolo R et al (2025) Guiding AI in radiology: ESR’s recommendations for effective implementation of the European AI Act. Insights Imaging 16:33. 10.1186/s13244-025-01905-x 39948192 10.1186/s13244-025-01905-x PMC11825415

