# Supplementary material for: Simulating workload reduction with an AI-based prostate cancer detection pathway using a prediction uncertainty metric
Source: Eur Radiol. 2025 Jun 7;35(12):7821–31. doi: 10.1007/s00330-025-11727-6 (PMC12634809; doi:10.1007/s00330-025-11727-6)
Supplement: Supplementary file 1 — Supplementary information [file 330_2025_11727_MOESM1_ESM.pdf]

Simulation Workload Reduction with an AI-based Prostate Cancer Detection Pathway using a Prediction Uncertainty Metric

ELECTRONIC SUPPLEMENTARY MATERIAL

A. Experiment 1: *Comparing meanUQ and varUQ: performance metrics*

	Center A (n=689)		Center B (n=723)		Center C (n=200)	
	meanUQ	varUQ	meanUQ	varUQ	meanUQ	varUQ
Certain cases	n=459 (66.61%)	n=224 (32.51%)	n=475 (65.70%)	n=247 (34.16%)	n=125 (62.50%)	n=62 (31.00%)
Youden threshold	0.32	0.094	0.29	0.087	0.31	0.037
AUROC	0.72	0.72	0.68	0.78	0.91	0.95
True positives	20.04%	27.23%	16.21%	24.29%	15.20%	25.81%
False positives	79.96%	72.77%	83.79%	75.71%	84.80%	74.19%
True negatives	59.37%	73.42%	58.30%	74.53%	51.35%	72.26%
False negatives	40.63%	26.58%	41.70%	25.47%	48.65%	27.74%

Table S1 provides an overview of the performance metrics in the certain cases selected using meanUQ or varUQ per center.

B. Experiment 2: Workload reduction: sensitivities at fixed specificity

Table S2 shows the performance of the semi-autonomous AI-based rule out pathway versus the radiologists' pathway performance at the non-inferior sensitivity margins of 0.05 and 0.10. Notably, when performing a non-inferiority analysis on the radiologists' sensitivity, the number of false-negative patients increases with an accepted margin. This increase in false-negative patients may result in long-term patient harm because a proportion of csPCa patients will not receive treatment.

	**center A (n=689)**		**center B (n=723)**		**center C (n=200)**	
**Radiologists (reference)**	58% specificity at 83% sensitivity		54% specificity at 86% sensitivity		78% specificity at 95% sensitivity	
**Rule out (%)**	Specificity (%) at 78% sensitivity (0.05 margin)	Specificity (%) at 73% sensitivity (0.10 margin)	Specificity (%) at 81% sensitivity (0.05 margin)	Specificity (%) at 76% sensitivity (0.10 margin)	Specificity (%) at 90% sensitivity (0.05 margin)	Specificity (%) at 85% sensitivity (0.10 margin)
10	58	61	56	57	73	78
20	59	61	59	61	73	78
30	61	63	58	63	75	79
40	61	63	58	65	76	80
50	62	66	56	65	76	81
60	56	66	53	63	80	81
70	53	65	52	63	80	82
80	59	66	56	66	81	83
90	61	66	53	63	80	84
100	61	65	54	63	75	83

Table S2. This table shows the performance of the semi-autonomous AI-based rule out pathway versus the radiologists' pathway performance at the non-inferior sensitivity margins of 0.05 and 0.10. In blue, the radiologists' specificity performances are shown (center A: 83% sensitivity and 58% specificity, center B: 86% sensitivity and 54% specificity, and center C: 95% sensitivity and 78% specificity). In green, the proposed AI-based rule out pathway specificity performances are shown that maintain or improve the specificity of the radiologists up to the first reduced specificity. For example, at a 0.05 non-inferior sensitivity margin, the performance of 10% AI model readings in center A is 78% sensitivity at 58% specificity versus the radiologists' performance of 83% sensitivity and 58% specificity.
